# Adolescent Profiles According to Their Beliefs and Affinity to Sexting. A Cluster Study

**DOI:** 10.3390/ijerph17031087

**Published:** 2020-02-08

**Authors:** Encarnación Soriano-Ayala, Verónica C. Cala, Rachida Dalouh

**Affiliations:** Department of Research Methods in Education, University of Almeria, 04120 Almeria, Spain; vcc284@ual.es (V.C.C.); rachidadalouh@ual.es (R.D.)

**Keywords:** sexting, adolescence, clusters analysis, Spanish, Moroccan

## Abstract

Sexting consists of sending, receiving, and distributing images of sexually suggestive content through electronic devices. This practice is one of the new ways of linking sex affectively through virtual environments, especially in adolescence. However, not all young people have the same relationship with the practice of sexting. This study of a sample of 603 Spanish and Moroccan adolescents residing in Andalusia analyzes beliefs towards sexting as part of a virtual sexuality and the perception of those who carry it out, defining profiles of affinity to sexting. The cluster analysis reveals the existence of three predominant profiles: adolescents who show a sexting-philia, perceiving it as a fun, flirty, and daring practice; sexting-phobes, who consider sexting to be characteristic of people, or attitudes, who are desperate, impolite, and conflicting; and a third ambivalent profile of people who appreciate the practice as something fun but conflicting. The majority discourse is one that presents a positive view of this phenomenon. Young people also recognize that sexting has some characteristics of virtual sexuality, such as a loss of privacy and a distance between virtual and real behavior. These findings allow us to deepen our understanding of the new practices of relationships and offer measures for the prevention of the associated risks.

## 1. Introduction

The rapid growth of social media is bringing about a series of transformations in the ways people relate and behave, particularly in the case of those who make up the first generation of digital natives: adolescents. The virtual experience, time spent on socialization through virtual networks, has become one of the main sources of behavior acquisition. For authors such as Berardi [1] or Sibilia [2], the changes that have occurred as a result of the use of digital technologies, due to their intensity and depth, have acquired the status of mutation. Anthropological mutation refers to the development of radical changes in sensitivity—in the ways people perceive, understand, relate to, and interpret the world. To be more specific, major changes have been noticed in seduction or in courtship and in the establishment of affectional–sexual bonds. An evolution has been identified towards more explicit and immediate, less ambiguous ways to establish contact between people than existing methods that did not entail the use of technology. In this process of virtualizing sexual–affectional relationships, of forming a “virtual sexuality”, among other things, we find the trend of sexting.

Sexting is one of the results of incorporating digital technologies into adolescent life. This practice has been defined as the sending, receiving, or forwarding of text, image, or video messages with sexual or suggestive content, which may include nudity or semi-nudity, through mobile phones, tablets, or similar devices [3,4,5,6]. The main motivation behind it is to seduce or please one’s partner, but it is also known to be used as a means of entertainment and a joke about third parties [7]. The prevalence of this trend is variable. One of the sources of variability lies in the definition used [8]. When more restricting definitions are used, which only consider sexting to occur when there are sexually explicit or nude images, we find that approximately 5%–28% of adolescents admit to sexting [9], whereas when broader definitions are used, which also recognize suggestive images, over 60% of young people admit to sexting [10].

The emergence of this trend and its collateral effects has caused a tremendous stir in some English-speaking countries, giving rise to what has become known as “sext panic”, a media discourse raising social alarm and a desire to control sexting due to the severity of some of the risks involved. Among the risks associated with sexting are child pornography, grooming, sextortion, or cyberbullying, which, in some cases, have led to the people involved committing suicide. Concern about sexting has generated debates as to how to understand and how to deal with it [11,12], including whether we need to take more prohibitionist and punitive measures that protect adolescents as vulnerable individuals [13] or merely educational measures, considering sexting to be just another part of adolescent development in the digital age [5,9,14], or whether, after a person engages in sexting, there is any sexual agency of adolescents.

### 1.1. Sexual Script Theory, Virtual Sexuality, and Sexting

In order to understand sexual–affectional ways of acting in virtual environments, it is useful to refer to sexual script theory [15,16]. This approach holds that sexual behavior is established from a series of social scripts or stories that guide our conduct, giving meaning to our feelings and body conditions. These scripts participate in the construction of desire and in the formation of sexual identities. According to this theory, individual behaviors are developed on the basis of three fundamental scripts: cultural, interpersonal, and intrapsychic, which feed into each other. The cultural script sets the standards that will govern the collective sphere of life, i.e., the representations, codes, and values that establish which sexual expressions are permitted and which are not. Different institutions are involved in the formation of this script: press and media, social media, peers, family, and religious institutions [16,17]. The interpersonal script regulates daily interactions and negotiations with other actors according to the wishes and expectations of each individual. The intrapsychic script refers to the internalization of social and cultural aspects that are articulated in the form of emotions, desires, fantasies, fears, or worries involved in a person’s sexual and affectional life, whether or not these are conscious aspects.

The formation of the sexual cultural script is established from the integration of different scripts in each of the social institutions, each of which has its own peculiarities. The internet has given rise to its own virtual sexual culture. For digital natives, the sexual cultural script lies somewhere in between the social norms in a non-digital environment (family, peers, religion, etc.) and the norms in the virtual environment. Different authors have characterized virtual sexuality with the following features: (1) wider possibilities for socialization, being able to choose, maintain, and discard numerous contacts at the same time, in a logic that is marked by consumption, and which can simultaneously come with social isolation [2]; (2) the construction of a new, improved virtual identity that is modeled around a mediatized outward appearance, in other words, one that is based on ideal models of perfection, success, coolness, beauty, and happiness, which, in some cases, comes with a dissociation between one’s real and virtual identities; (3) greater access to sexual information (pornography, other sexual identities, global references, new practices) [18], which leads to a multiplication of possible sexual identities, constant hyperstimulation of the libido, and hypersexualization of image and behavior according to gender and sexual identities—to this effect, there are authors who hold that there is a certain tension between the emancipation and extension of sexual freedoms and the processes of objectification and pornification of a generation in which individuals acquire the role of fetishized sexual objects; (4) the absence of physical contact with the other, preventing the consolidation of strong affectional bonds, which leads to a depersonalization of relationships and the formation of loose bonds; (5) digital narcissism or virtual ego (ego boost) strengthened by the virtual boost created by ratings logic (popularity through likes), which generates social hierarchies between peers in line with ratings [19,20]; (6) a perception of virtual sexuality as safe and protected, hidden behind the screen [21]; (7) a new paradigm of intimacy: “extimacy”, where the boundaries between public and private become blurred, as intimate relationships are externalized [22].

Cultural scripts are not universal; they are contextual, and they vary according to age, origin, gender, social class, environment, and social media, establishing different codes of practice (Bourdieu). In this way, behavioral patterns and discourses in the virtual environment differ between adolescents and adults, between gender identities, etc. [23]. Most qualitative studies into adolescent discourses regarding sexting reveal two positions in this respect [24,25]: those who assume the dominant discourse, who accept the requirements of virtual sexuality, and those who object and who define their positions as a rejection of virtual sexuality or in line with traditional sexual culture offline. Lippman and Campbell [26] claim that most of the adolescents who were interviewed approve of the standard culture of sexting, and they continue to sext, even with people who have not given their consent, despite being aware of the risks. However, there is a minority of adolescents who feel that sexting could damage their reputation.

### 1.2. Objectives

The objective of this study focuses on identifying the beliefs existing around the trend of sexting in Spanish and Moroccan adolescents in the south of Spain. By establishing and classifying Spanish and Moroccan adolescents’ relationships with sexting, this study enables us to define the main profiles of such adolescents based on their attraction to or rejection of sexting. It also enables us to sketch out some basis for their virtual sexual scripts.

Some of the hypotheses guiding this study coincide with the characteristics given about virtual sexuality: a perception of loss of privacy will be identified, as well as a dissociation between real and virtual identities. With regard to the profiles on sexting, one would expect there to be two: one mostly in favor of it and another, minority group, who reject the practice. The favorable profile will mostly be made up of Spanish women and Moroccan men, these being the two groups that most claim to engage in sexting.

Knowing the beliefs of different sociocultural groups towards sexting and different profiles can help design inclusive educational programs and thereby contribute to the achievement of the Millennium Sustainable Development Goals for inclusive quality education (objective 4.7) and universal sexual health education (objective 3.7) [27,28].

## 2. Materials and Methods

### 2.1. Participants

The sample was made up of 642 adolescents, 79.8% of which were Spanish and 20.2% were 1.5-generation Moroccan immigrants (born in Morocco but socialized in Spain). The average age of the participants was 14.43 (SD = 1.63), and the age range was between 12 and 17 years old. These sex and origin variables define the four socio-cultural groups in this study: 254 Spanish women (SW), 258 Spanish men (SM), 67 Moroccan women (MW), and 63 Moroccan men (MM). A total of 85.8% of the Spanish women and 85.2% of the Spanish men were Catholic, whereas 98.8% of the Moroccan women and 95.8% of the Moroccan men were Muslim. There were nine cases of Spaniards who converted to Islam and two cases of Moroccans who practice Catholicism, which suggests the existence of mixed marriages. The rates of atheism were low, with levels of 11% in the Spanish participants and 0% in the Moroccan participants. A total of 51.4% of the adolescent Moroccan men, 22.5% of the adolescent Moroccan women, 20.4% of the adolescent Spanish men, and 29.7% of the adolescent Spanish women said they had a partner at the time of the data collection. Regarding sexual relations, 2.5% of the Muslim women said they had had sex, compared with 51.7% of the Moroccan men. The figures for the Spanish men and women were similar, with one out of four claiming to have had sex (25%).

### 2.2. Procedure

A prescriptive and descriptive cross-sectional study was carried out, for which probabilistic cluster sampling was used to select eight secondary education centers located in underprivileged areas, both rural and suburban, in south-east Spain (in the provinces of Almería, Murcia, and Malaga), which are characterized by settlements of economic immigrants, specifically in areas in which the percentage of the foreign population is over 25%. We were authorized to access these centers by the Department of Education. The principals of each center authorized the data collection after obtaining parental permission. The scales were applied to the students by the project researchers in their classrooms in the presence of their teachers.

### 2.3. Instruments

For this study, two sub-scales from the Sex and Tech survey (The National Campaign to Prevent Teen and Unplanned Pregnancy) [29] were used. With the total scale, we followed a process of translation and back-translation, and with the help of experts in sexology and interculturality, the content was adapted and validated.

The Sex and Tech survey (The National Campaign to Prevent Teen and Unplanned Pregnancy, 2008) defines sexting as “sending or receiving sexually suggestive content” via technological devices. We selected two sub-scales for our study. The first includes the items related to the actions of friends sending, posting, or sharing suggestive images of themselves or of others; the difference between sending and sharing in real life and in a virtual life; and the seriousness of the facts in question. This sub-scale has a Cronbach’s alpha coefficient of α = 0.703. In the second scale, the adolescents were asked to state their degree of agreement/disagreement with adjectives used in the sub-scale to describe people who send provocative messages and images. The adjectives in question were the following: conceited, rude, conflictive, stupid, bold, confident, cool, desperate, fun, immature, and flirty. To find the total description of the scale, the negative terms were recoded to make them positive. The reliability of this sub-scale is α = 0.739.

### 2.4. Data Analysis

For the first scale, descriptive and inferential calculations were made through logistic regressions using the SPSS 24 statistics software package (IBM, Chicago, IL, USA). To consider the results of the logistic regressions, the Hosmer–Lemeshow inference test was applied, and when the test gave a non-significant result (*p* > 0.05), the predictive model was assumed, and the results evaluated. In this test, the dependent and independent variables (country of origin and sex) are dichotomous. In the dependent variables, a value of 0 is given to the category of reference, and a value of 1 is assigned to the category we consider of risk. In the independent variable, the category of reference was considered to be a man (0) for sex and Moroccan (0) for country of origin, for example. For the second scale, a k-means cluster analysis was carried out to determine the profiles among the adolescents in the study.

### 2.5. Ethical Issues

The participants received information about the research study and about the academic institution that is responsible for the study. Informed consent was given, expressing the voluntary nature of participating in the study as regards the student, the right to leave the study, and the anonymous and confidential nature of the data collected.

## 3. Results

### 3.1. Beliefs about Sexting in Adolescents

In the seven logistic regression models, we included the country of origin and sex as independent variables to determine which predictors represented a variance in each one of the criterion variables (Table 1). The predictive capacity of the country of origin had a significant effect on the assertion that “People my age are ‘bolder’ in their messages and in sending sexy photos or videos than they are in real life” (OR = 2.05), and it was twice as probable that Spanish people think that adolescents are bolder in the virtual world when engaging in sexting than Moroccans. The opposite occurs in the statement “Sending suggestive messages and images of oneself can have serious negative consequences” (OR = 0.28); given that the value was lower than 1 (1/0.28 = 3.57), it was 3.57 times more probable that the risk of sexting is perceived by Moroccans than by Spanish people. In the statement “I am ‘bolder’ when it comes to sending suggestive messages and images than I am in real life” (OR= 0.51, 1/0.51 = 1.96), the results indicate that it is almost twice as probable that this behavior is shown more by Moroccan adolescents than Spanish adolescents. As regards the prediction of sex, the results found that the statement “I am ‘bolder’ when it comes to sending suggestive messages and images than I am in real life” (OR = 0.72, 1/0.72 = 1.39) is 1.39 times more probable in men than in women. For the rest of the statements presented to the adolescents in the study, similar responses were found between men and women and between Spanish and Moroccan participants.

### 3.2. Description by Adolescents of People Who Engage in Sexting: Profiles

For the purposes of defining profiles related to the description made by adolescents of people who engage in sexting, cluster sampling analysis technique was used. This technique forms groups based on similarities with respect to a series of characteristics, obtaining internally homogeneous groups that are heterogeneous between each other. Given that our sample was made up of 642 adolescents, we chose a non-hierarchical procedure known as k-means clustering. This method is considered to be efficient, because it does not calculate the distance between all the pairs of cases and because it does not require the resulting groups out of so many cases and intervening variables to be visually established. Nevertheless, it does have the disadvantage of not defining the number of groups. This must be done by the researcher and then included in the analysis.

In this study, we carried out multiple classification analyses to prioritize the data parsimony criteria, in other words, we endeavored to identify the solution that was most repeated. The results revealed the following (Table 2): (1) The number of groups that met the law of parsimony was 3. (2) The ANOVA test of the cluster study showed significant differences (*p* = 0.000) in all variables. 3) To verify the effectiveness of the clustering into the 3 groups, we applied the Kruskal–Wallis comparison test, as the data did not fit a normal distribution, to the total description of the people who engage in sexting, with a Chi-square = 300.156 and *p* = 0.00.

**Figure 1:**
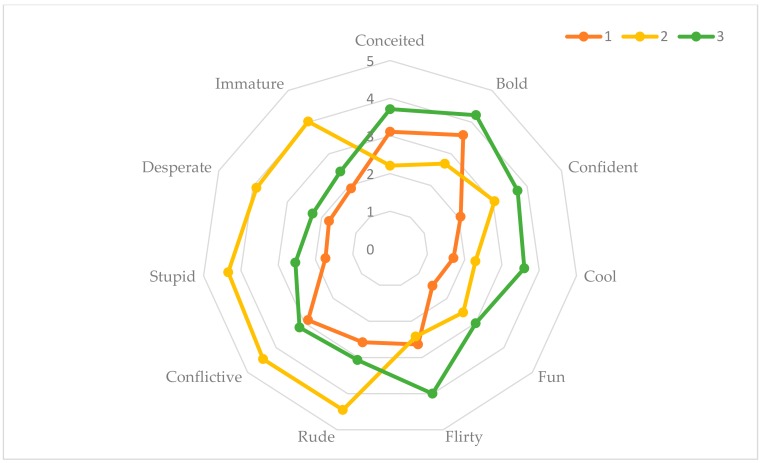
Three identified profiles or clusters according to the rating given by the students as regards sexting.

According to the data from the overall sample, the following profiles were, as can be seen in Figure 1:

Cluster or profile 1. This is an ambivalent profile. It is a weak negative with some positive traits. The members of this profile describe people who engage in sexting as conceited, bold, and conflictive.

Cluster or profile 2. This is a clearly negative profile towards people who engage in sexting, describing them as rude, stupid, desperate, conflictive, and immature.

Cluster or profile 3. This is a profile with a positive value, in which people who engage in sexting are seen as bold, confident, cool, fun, and flirty.

Profile 1: Ambivalent. It represented 26% of the participants; the mean was 2.32 and was below the scale’s mean score (3.021). The standard deviation was not high (0.39); it was below the overall score (0.597); therefore, there was greater homogeneity in the individuals’ responses.

Profile 2: Negative. It represented 28.3% of the participants. The mean score for this profile was 3.35, which was above the mean for the overall description of the total participants (3.01). There was greater dispersion in the responses than in profiles 1 and 3, with the standard deviation being 0.51.

Profile 3: Positive. This profile represented 45.7% of the participants. The mean score was 3.21, above the mean (3.02) of the overall description of all participants. The standard deviation was 0.38, which indicates that the participants’ responses were the least dispersed.

### 3.3. Study of The Profiles for Spanish and Moroccan Adolescents

After studying the profiles of Spanish and Moroccan adolescents, significant differences were found in the three profiles for the two groups (Table 3). The highest mean score was obtained by Spanish adolescents in profile 2, who were clearly negative towards people who engage in sexting, describing them as rude, stupid, desperate, conflictive, and immature, with the only significant difference from Moroccan adolescents being found in this profile. Profile 3 obtained the second highest mean score for Spanish adolescents. The members in this profile had a positive perception of people who engage in sexting.

The Moroccan participants obtained slightly lower mean scores than did the Spanish participants. It is worth pointing out that Moroccans obtained the highest mean in profile 3, with a positive evaluation of people who engage in sexting.

## 4. Discussion

### 4.1. Beliefs about Sexting and Virtual Cultural Script

The results related to beliefs about sexting reveal that half of the adolescents in the study believe that young people of their age engage in sexting. These figures are found within the ranges acknowledged in the work by Barrense-Dias et al. [10], with similar figures to those given in other studies focusing on passive sexting, and higher than those in other Spanish studies [30,31]. The fact that studies asking about sexting in peers give higher figures than those that focus on sexting that has actually been done is compatible with a bias in the self-recognition of the practice itself, as has been shown in other issues about sexuality in adolescence [32].

With regard to those aspects of sexting as a part of virtual sexuality, such as a loss of privacy, dissociation between virtual and real identity, or a perception of the internet as a safe space, we can see that these issues are widely internalized by many of the participants, as we stated in our first hypothesis. Half of young people, regardless of their origin or gender, believe that there is pressure to engage in sexting [25]. For authors such as Walrave et al. [33], the source of pressure comes mainly from friends and peers, although it can also come from one’s partner. Although they may be statistically non-significant, the results show that Spanish women and Moroccan men feel the most pressure. In previous studies, both groups have been revealed to be the most active on social media and those who engage in sexting the most [7]. This coincidence enables us to think about mechanisms through which more time spent in the virtual world is associated with a greater perception of pressure to engage in sexting.

Another widely held belief among young people (approximately half of the adolescents in our study) is that women are the ones who must protect their own privacy, which coincides with research studies that associate being a woman with greater vulnerability and pressure on social media [34,35,36]. The analysis of sexual scripts by gender in adolescence describes how men take on more of a predator and consumer role, whereas women act more like sexual bearers [37], maintaining a double sexual standard in the virtual environment. Setty [24] analyzes the discourses about sexting in adolescents according to their type of masculinity. For men who come under a Western hegemonic masculinity, sexting is perceived as more acceptable, less risky, and less concerning than it is for women. They describe masculine sexuality as sexually desiring, hormonal, inevitable, and uncontrollable and sexting as a logical consequence of this [38]. This masculinity rewards those who get and distribute images of young women, hailing them as “heroes”, which allows them to accumulate “power and recognition” among their peers. This heroification arises even more when the images are of “decent or good” young women. However, there are also critics in what has come to be known as alternative masculinities, who criticize sexting without mutual consent.

As regards the difference between behaviors in the virtual environment and in real life, half of the participants admit to behaving differently in the two settings. Young Moroccan adolescents, with a probability of almost double that of Spanish adolescents, perceive themselves as bolder or more daring online than in real life. To explain the distancing between the virtual self and the real self, two hypotheses were posed: that of fragmentation and that of unity of identity [39]. The fragmentation hypothesis holds that one’s identity is split—a virtual avatar is created that is dissociated from oneself, and the online self is generally a consciously improved version of oneself. The unity hypothesis, on the other hand, holds that, due to the characteristics of virtual spaces, young people have more opportunities to contact a lot of people, which enables them to modify their identity in a much deeper way, both online and offline. Both hypotheses assume the existence of different online and offline cultural norms, to which young people must adapt or transform. Furthermore, the greater degree of real/virtual dissociation shown by the Moroccan group compared with the Spanish group can be explained on the basis of a greater degree of insecurity in their real identity. Studies show that Moroccan adolescents have lower levels of adaptation than other immigrant adolescents and fewer socio-emotional skills than native Spanish adolescents [40,41], which may have a negative influence on their capacity to interact with others in the real world, thus fostering their online/offline split.

Lastly, with regard to the degree of their awareness of the risks involved in online activity, the Moroccan participants were revealed to be significantly more aware of these risks than the Spanish participants. To this effect, the study carried out by Pagán et al. [42] in Murcia shows that immigrant adolescents are the group that accepts the most unknown contacts online. A possible explanation for this difference, therefore, is that Moroccans have greater contact or experience with risk situations, which enables them to be more aware of the dangers online. Likewise, the different notions of privacy and intimacy existing in individualist societies, such as Spain, and in collectivist societies, such as Morocco, may also be relevant.

The set of beliefs revealed by the young people in this study fits with the characteristics of the aforementioned virtual sexuality. This reinforces the idea of a transition in the sexual cultural script, from a non-digital to a virtual one. This new or hybrid sexual script seems to change some traditional gender sexual stereotypes (women sexual empowerment) but maintain normative masculinity and the gender imbalance [43,44].

### 4.2. Profiles on Sexting: Positive, Negative, and Ambivalent

In comparison with the second initial hypothesis that predicted the existence of two essential profiles regarding an affinity to sexting (acceptance and rejection), the cluster study revealed three main profiles: ambivalent, negative, and positive. The proportion of adolescents in each group clearly favored the positive profile. Almost half of the participants (46%) fell into this group, regarding the practice of sexting as bold, safe, cool, fun, and flirty. The findings of this study coincide with those carried out by other authors [25,26] insofar as they hold that the majority and dominant discourse among young people is favorable to the practice of sexting and consequently to a virtualized sexuality. Some studies have focused on characterizing those people who show a more favorable attitude towards sexting with the following characteristics: higher levels of online disinhibition and a greater degree of objectification of women [36], greater impulsivity [45], and low socioeconomic level.

The second identified profile was that of those who maintain an ambivalent view (26%), regarding people who engage in sexting as bold and flirty, while at the same time considering them to be conflictive, integrating aspects from both the positive and negative profiles. In previous qualitative studies, the results showed pretty widespread ambivalent positions among young people [46]. This ambivalence may be also a reflection of the transition and contradiction processes between the traditional sexual cultural scripts (face-to-face) and the new virtual scripts. In the same way, the media discourse from families and safety and security bodies contradicts the practice, while fashion and the media are favorable. A coexistence between desire and rejection is generated from the synthesis of the discourse. In the case of women, ambivalence has also been explained on the basis of a new female empowerment discourse (also in the virtual environment) and the traditional elements of the dominant culture of criminalizing female sexuality [47].

Lastly, there is one group that openly rejects sexting (28%), considering it to be a practice that is typical of rude, stupid, desperate, conflictive, and immature people. Critical views about sexting have been recognized as characteristics of alternative adolescence, being more respectful and more ethical towards sexuality [24], but they also coincide with the prevailing legal and academic theoretical framework that understands sexting as a new type of deviant sexual behavior in youth, being associated with risks to one’s health as well as to immoral sexual behavior [5]. It would be useful to delve deeper into the discourse regarding the rejection of sexting to understand if it is linked to a more restrictive, censoring component or to a new sexual ethic based on maintaining intimacy and consensus.

The transcultural analysis of the identified profiles shows a similar proportion of Moroccan and Spanish individuals per profile, such that there is no clear sign of a more or less negative view than the overall attitude spread over the groups of origin. However, what we can see is that the mean scores per profile are lower in the Moroccan group, being statistically significant in the negative profile. This means that those young people who are born in Morocco with a sexting-phobic view rate those who engage in sexting comparatively worse. Just as they obtained the highest mean in that category, they also achieve it in profile 3 with a positive evaluation of people who engage in sexting. This implies that young Moroccan people have a more polarized view of sexting than Spanish adolescents.

## 5. Conclusions

In conclusion, this study recognizes the existence of beliefs associated with sexting that are linked to the conceptualization of a virtual sexuality, such as a loss of intimacy, feeling of pressure, and separation as regards offline behavior. The formation of new virtual affectional practices requires the traditional sex education paradigm to be redefined, integrating privacy in the digital age, free, conscious and critical decision-making, and the establishment of consensual relations as key aspects in all programs.

Furthermore, this study recognizes three profiles of acceptance, ambivalence, and rejection of sexting. The profile that accepts sexting is the majority profile. Education programs aimed at the group of Moroccan origin in areas of cultural diversity must take this on board as their starting point. It is not enough to implement a prevention scheme that warns of the possible risks; this must be accompanied by affective–sexual development in the virtual environment that is adapted to each of the three recognized profiles, which can be associated with different risks and problems. It is necessary to move forward with strategies adapted to these profiles that, in turn, integrate a gender perspective that is also sensitive to origin.

## Figures and Tables

**Table 1:** Beliefs about sexting in Spanish and Moroccan adolescents. SW: Spanish women; SM: Spanish men; MW: Moroccan women; MM: Moroccan men.

Beliefs about Sexting	SW *n* (%)	SM *n* (%)	MW *n* (%)	MM *n* (%)	Country Sex			
					OR β CI 95%		OR β CI 95%	
There is pressure among people of my age to post sexy personal photos or videos online.	177 (52.5)	160 (47.5)	38 (48.1)	41 (51.9)	0.256	1.29 0.868–1.92	0.18	1.19 0.863–1.66
Girls have to worry about the privacy of their suggestive messages and images more than boys.	223 (52.1)	205 (47.9)	53 (50)	53 (50)	0.156	1.16 0.70–1.94	0.323	1.38 0.90–2.11
People my age are “bolder” in their messages, sending sexy photos or videos, than they are in real life.	241 (52.1)	222 (47.9)	61 (52.1)	56 (47.9)	0.721	2.05 *	241 (52.1)	222 (47.9)
Sending suggestive messages and images of oneself can have serious negative consequences.	9 (33.3)	18 (66.7)	10 (47.6)	11 (52.4)	−1.25	0.28 ** 0.15-0.52	0.50	0.60 0.32–1.10
My friends have sent sexy photos or videos to other people.	220 (50.5)	216 (49.5)	55 (48.7)	58 (51.3)	−0.191	0.82 0.45–1.49	−0.22	0.97 0.61–1.54
My friends have posted sexy personal photos or videos online.	166 (47.4)	184 (52.6)	49 (51.6)	46 (48.4)	−0.24	0.78 0.50–1.22	−0.23	0.78 0.55–1.11
I am “bolder” when it comes to sending suggestive messages and images than I am in real life.	124 (46.4)	143 (53.6)	43 (48.3)	46 (51.7)	−0.67	0.51 *** 0.33-0.76	−0.32	0.72 * 0.52–0.99

* *p* < 0.05; ** *p* < 0.01; *** *p* < 0.00.

**Table 2:** Number of cases per cluster and distance between the cluster centers.

No. of Cases	Cluster	Distances between the Cluster Centers		
		1	2	3
160	1		5.094	3.573
177	2	5.094		4.630
272	3	3.573	4.630	

**Table 3:** Means, standard deviation, and contrasts using the Kruskal–Wallis and the Mann–Whitney U tests between Spanish and Moroccan adolescents.

Profiles	Spanish			Moroccan			Contrast	
	N	M	SD	N	M	SD	U	*p*
**Profile 1**	128	2.33	0.39	10	2.14	0.41	469.5	0.160
**Profile 2**	130	3.37	0.51	22	3.10	0.41	997.5	0.023*
**Profile 3**	193	3.20	0.39	44	3.16	0.32	3850	0.358
	Kruskal–Wallis in the 3 profiles Chi-square = 232.99; *p* = 0.000			Kruskal–Wallis in the 3 profiles Chi-square = 24.93; *p* = 0.000				
	Mann–Whitney U profiles Profiles 1-2; U = 635; *p* = 0.000 Profiles 1-3; U = 1156.5; *p* = 0.000 Profiles 2-3; U = 10471.5; *p* = 0.01			Mann–Whitney U profiles Profiles 1-2; U = 6; *p* = 0.000 Profiles 1-3; U = 2.5; *p* = 0.000 Profiles 2-3; U = 437.5; *p* = 0.525				
